# Culture medium influences detectability of antimicrobial effects of autologous platelet concentrates against anaerobic periodontal pathogens

**DOI:** 10.1186/s12903-026-08768-8

**Published:** 2026-06-02

**Authors:** Ellen E. Jansen, Andreas Braun, Patrick Jansen, Georg Conrads

**Affiliations:** 1 Department of Operative Dentistry, Periodontology and Preventive Dentistry, Rheinisch-Westfälische Technische Hochschule (RWTH) University Hospital, Aachen, 52074 Germany; 2 Division of Oral Microbiology and Immunology, Department of Operative Dentistry, Periodontology and Preventive Dentistry, Rheinisch-Westfälische Technische Hochschule (RWTH) University Hospital, Aachen, 52074 Germany

**Keywords:** Blood-derived biomaterials, Autologous platelet concentrates, Agar diffusion assay, Culture medium, Diffusion-based testing, Inhibition zone detectability, *Porphyromonas gingivalis*, *Prevotella intermedia*

## Abstract

**Background:**

Autologous platelet concentrates (PRP, PRF, i-PRF) represent blood-derived biomaterials that have been reported to exhibit antimicrobial activity against oral microorganisms in vitro. In diffusion-based assays, visualization of inhibitory effects depends not only on bacterial growth but also on matrix-dependent diffusion behavior within the surrounding culture medium, particularly those complex media used for fastidious anaerobic species. However, it remains unclear whether commonly used agar media provide comparable assay environments for evaluating APC-derived antimicrobial effects. This feasibility study therefore investigated whether PRP, PRF and i-PRF generate measurable inhibition zones on different agar matrices and whether medium selection influences inhibition detectability and measurable zone expression in diffusion-based assays.

**Methods:**

Inhibitory effects of PRP, PRF and i-PRF against *Porphyromonas gingivalis* and *Prevotella intermedia* were tested on Brucella blood agar (BBA), Mueller–Hinton blood agar and Tryptic Soy Agar supplemented with blood (TSASB). Only measurable inhibition zones were included in quantitative analyses, while detectability outcomes were assessed separately.

**Results:**

BBA produced significantly larger measurable inhibition zones for *P. gingivalis* compared with TSASB (*p* = 0.0006; Cliff’s δ = 0.92). For *P. intermedia*, BBA and MHB showed comparable inhibition patterns (*p* = 0.77), whereas TSASB yielded no measurable inhibition zones. Inhibition effects were detectable for all investigated platelet preparations. Exploratory comparisons between PRP, PRF and i-PRF across culture media did not reveal statistically significant differences under the present experimental conditions.

**Conclusion:**

Agar composition represents an active experimental matrix in diffusion assays and critically shapes inhibition zone expression, particularly for strict anaerobic periodontal pathogens. Culture medium selection therefore constitutes a key methodological variable in antimicrobial evaluation of APCs, and negative findings in diffusion assays should be interpreted cautiously within the context of the underlying assay environment.

## Introduction

 Autologous platelet concentrates (PRP, PRF and i-PRF) are widely used biological preparations that have been reported to exhibit antimicrobial activity against oral microorganisms in vitro [1]. In addition to growth factors, autologous blood derivatives contain antimicrobial peptides and host defence molecules, such as defensins and cathelicidins, which may contribute to inhibitory effects against bacteria [2–4].

A large number of experimental studies demonstrate the antimicrobial activity of PRP, PRF and i-PRF against periodontopathogenic bacteria, including *Porphyromonas gingivalis* (*P. gingivalis*) and *Prevotella intermedia (P. intermedia)* [1, 5–7]. Systematic reviews consistently highlight that discrepancies in antimicrobial activity arise largely from methodological heterogeneity in vitro, including variations in culture media, inoculum, incubation conditions and preparation protocols [8–10].

From a microbiological perspective, antimicrobial diffusion assays require two prerequisites: robust and homogeneous bacterial growth, and sufficient diffusion of inhibitory components within the agar matrix [11]. Inhibition zone formation therefore represents a diffusion-dependent system readout rather than a direct measure of intrinsic antimicrobial potency. If either condition is not fulfilled, inhibition may not become visible despite underlying biological activity, resulting in false-negative or non-detectable outcomes.

Bacterial growth performance and diffusion behaviour are strongly influenced by culture medium composition, including nutrient content, pH, and supplementation. For this reason, the European Committee on Antimicrobial Susceptibility Testing (EUCAST) and the Clinical and Laboratory Standards Institute (CLSI) recommend standardised Mueller-Hinton agar (with defined additives for demanding pathogens) for disc diffusion and provide narrowly defined target values for inhibitory site diameters via quality control Tables [12–15]. Even within different Mueller-Hinton brands, differences in pH and cation contents lead to measurable deviations in the inhibition zones [14]. In diffusion-based assays, the culture medium therefore represents an active experimental matrix rather than a neutral substrate. For autologous platelet concentrates, however, comparable standardization does not yet exist. Consequently, studies investigating identical platelet preparations may report divergent antimicrobial results when experiments are conducted under different culture conditions.

Fastidious anaerobic oral microorganisms represent a particular methodological challenge. *P. gingivalis* and *P. intermedia* are among the most important periodontopathogenic microorganisms of high clinical significance and are consistently identified in the literature as key pathogens in the aetiology of periodontitis [16, 17]. *P. gingivalis* is a strictly anaerobic pathogen highly adapted to the periodontal environment, whose growth and virulence are significantly influenced by oxygen tension, redox potential and nutrient profile of the medium [18, 19]. The same applies to *P. intermedia*, which, as an important representative of the “orange complex” microbiota, also places pronounced demands on anaerobic conditions and iron-containing growth factors [20]. Under suboptimal culture conditions, growth may become patchy or insufficient, which precludes reliable interpretation of inhibition assays.

There are currently no standardized protocols for the evaluation of autologous platelet concentrates, such as those defined for antibiotics by EUCAST. The existing in vitro studies of PRP/PRF/i-PRF use a variety of different agar media, including blood agar, modified brain-heart-infusion agar, Mueller-Hinton derivatives, and specialty anaerobic media. Experimental approaches vary considerably and include diffusion assays, liquid culture models, and biofilm systems [8, 9, 21]. This suggests that experimental conditions, including the choice of culture medium, may contribute to variability in inhibition zone detectability and size [5, 7]. Such variability suggests matrix-dependent modulation of inhibition zone expression rather than purely biological differences between APC formulations.

To date, there is no systematic study that examines the influence of agar selection on inhibition zone visibility for autologous platelet preparations. The present feasibility study therefore investigates how different agar media influence bacterial growth compatibility and the detectability of inhibition zones generated by PRP, PRF and i-PRF. The study specifically addresses matrix-dependent effects in diffusion-based evaluation of blood-derived biomaterials.

## Materials and methods

This methodological in vitro feasibility study investigated the influence of different culture media on inhibition zone detectability and measurable inhibition zone expression. Focus was placed on the two anaerobic key pathogens *P. gingivalis* and *P. intermedia*, which are known to be difficult to cultivate.

The protocol was approved by the local ethics committee (CTC-A-Nr.21–329, EK 379/21, RWTH Aachen University) and conducted in accordance with the Declaration of Helsinki [22]. All participants provided written informed consent, and samples were anonymized prior to analysis.

### Microorganisms and preparation of bacterial suspensions

Bacterial suspensions of *P. gingivalis* (ATCC 33277^T^) and *P. intermedia* (ATCC 25611^T^) were prepared from fresh cultures and adjusted to McFarland standard 0.5 (1.5 × 108 cells/ml). For inoculation, 200 µL of the standardized suspension (corresponding to approximately 3 × 107 CFU per plate) were applied and evenly distributed using a Drigalski spatula to ensure formation of a dense and homogeneous bacterial lawn. This increased inoculum volume was selected to improve growth consistency and inhibition zone detectability for fastidious anaerobic species, as preliminary experiments with lower volumes (e.g., 100 µL) resulted in insufficient or patchy growth. Plates were pre-incubated for 4 h at 37 °C under anaerobic conditions prior to application of platelet concentrates. This step was applied identically for both species to facilitate initial bacterial adaptation and establishment of a uniform early growth phase on the agar surface. The selected duration was based on preliminary experiments demonstrating improved lawn formation and inhibition zone detectability compared with shorter or omitted pre-incubation. Bacterial suspensions were prepared from standardized stocks generated from a single culture and stored in aliquots at − 80 °C. For each experiment, aliquots were thawed to ensure consistent starting conditions across experimental runs.

### Culture media

Three agar media were investigated: Brucella blood agar (BBA), Mueller–Hinton blood agar (MHB), and tryptic soy agar supplemented with sheep blood (TSASB). Media were selected to reflect commonly used diagnostic agars, including systems suitable for fastidious and anaerobic organisms. Based on preliminary experiments and species-specific assay performance, culture media were applied differentially for the investigated microorganisms. TSASB and BBA were tested for both species. In contrast, Mueller–Hinton blood agar did not provide sufficiently robust or consistent bacterial growth for *P. gingivalis* under the present experimental conditions and was therefore not included for this species, but was used for *P. intermedia*. Commercial TSASB plates (Thermo Scientific, PB5012A) and commercial MHB plates (Thermo Scientific, PB5007A) were used. BBA was prepared in-house as required. No physicochemical modification of the agar matrices was performed. For in-house preparation, BBA was prepared using 14 g Brucella broth (BD BBL 211088) and 9 g agar in 500 mL distilled water. After autoclavation, the medium was cooled down to 50 °C and supplemented with 5 mL Vitamin K1–Hemin solution (BD 212354) as well as 5% sterile, defibrinated sheep blood.

### Platelet concentrates

Autologous platelet concentrates were prepared from five systemically healthy, non-smoking volunteers without systemic antibiotic therapy within the preceding six months. Additional exclusion criteria included pregnancy and known coagulation disorders. From each donor, 30 ml of venous blood was collected by antecubital venipuncture using a sterile butterfly cannula into sterile tubes. PRP was prepared using citrate tubes with separation gel (Vi PRP-PRO tubes, Vi Medical GmbH, Bingen am Rhein, Germany) and centrifuged in an Eppendorf 5810R centrifuge (Eppendorf AG, Hamburg, Germany) at 1200 × g for 7 min, resulting in separation of platelet-rich plasma. PRF and i-PRF were prepared using Choukroun’s A-PRF™ and i-PRF™ glass tubes (mectron Deutschland Vertriebs GmbH, Cologne, Germany). For PRF preparation, blood was centrifuged at 700 × g for 8 min, after which the PRF clot was obtained. For i-PRF preparation, blood was centrifuged at 400 × g for 3 min, resulting in a liquid platelet-rich upper layer. All concentrates were produced according to the manufacturers’ instructions and used within 30 min after preparation.

### Plate inoculation

**Fig. 1:**
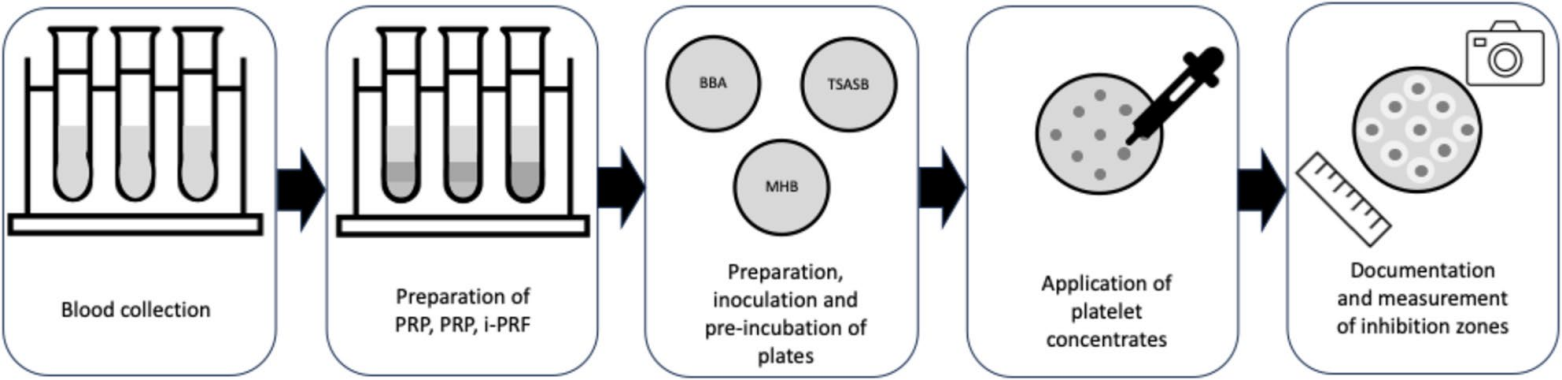
Schematic overview of the diffusion-based inhibition assay used to evaluate culture medium–dependent inhibition zone detectability of PRP, PRF, and iPRF against *P. gingivalis* and *P. intermedia*

A volume of 5 µL of each platelet concentrate (PRP, PRF, i-PRF) was pipetted onto the surface of the inoculated agar plates. Application volumes were standardized across all media to ensure comparable diffusion conditions. Plates were sealed and incubated under anaerobic atmospheric conditions for 72 h. A 0.9% sodium chloride solution (NaCl; B. Braun SE, Melsungen, Germany) served as the negative control, while chlorhexidine (2%, clinical stock solution, University Hospital RWTH Aachen, Aachen, Germany) and amoxicillin discs (10 µg; Oxoid Ltd., Basingstoke, UK) were included as positive controls. Chlorhexidine was included to provide a clinically relevant antiseptic reference with clearly delineable inhibition zones. Amoxicillin served as a standard antibiotic control; however, due to its strong antibacterial activity, it frequently produced very large inhibition zones under the present experimental conditions, limiting its suitability for comparative interpretation. Positive controls consistently produced clearly visible inhibition zones in all experiments. Due to their substantially different inhibition profiles, control data were not included in the quantitative analysis. Experiments were conducted over multiple days, with 1–2 donors processed per day. Agar media, including in-house prepared BBA, were produced in multiple batches during the study period. A schematic overview of the experimental workflow is shown in Figure 1.

### Assessment of inhibition zones

Due to the slow growth characteristics of the investigated anaerobic species, plates were evaluated at 24, 48, and 72 h, with final assessment based on the 72 h time point to ensure reliable growth and inhibition zone delineation. Detectability was defined as the presence or absence of a measurable inhibition zone. Non-measurable (NN) outcomes were recorded when no clearly delineable inhibition zone could be identified or when bacterial lawn formation was insufficient to allow reliable interpretation. Zone diameters (mm) were measured retrospectively using FIJI (FIJI – Fiji Is Just ImageJ, version 2.16.0/1.54p, ImageJ2 framework; ImageJ.net/Fiji; based on ImageJ, National Institutes of Health, Bethesda, MD, USA) by averaging two perpendicular measurements and results were documented digitally. Measured inhibition zones reflect the interaction between inhibitory components and matrix-dependent diffusion properties.

To assess measurement reliability, approximately 11% of inhibition zones were independently re-measured by a second blinded examiner.

### Statistics

Inferential statistics were applied in addition to descriptive analysis (mean, median, standard deviation, 95% confidence interval). Technical replicates were averaged per donor, preparation and microorganism before statistical analysis. Non-measurable outcomes (NN) were excluded from quantitative inhibition zone size analysis, as no valid numerical measurement can be obtained in the absence of a clearly defined inhibition zone. Instead, NN outcomes were analyzed separately as part of detectability assessments, representing a distinct methodological endpoint. Given the methodological focus of the study, inhibition zone measurements were analyzed at the assay level to explore matrix-dependent effects under controlled conditions. This approach treats each experimental condition as an independent observation; however, potential dependencies between measurements derived from the same donor cannot be fully excluded and should be considered when interpreting the results. Statistical comparisons were performed to assess whether culture media systematically influence inhibition zone expression. Mann–Whitney U tests were performed to compare inhibition zone distributions between culture media, and Cliff’s δ was calculated as an effect size measure to quantify the magnitude of medium-dependent differences. Pearson correlation analysis was used to assess inter-observer agreement for inhibition zone measurements. A two-sided *p* value < 0.05 was considered statistically significant.

All statistical analyses were performed using IBM SPSS Statistics 30 (IBM Corp., Armonk, NY, USA). No a priori power calculation was performed because of the exploratory feasibility design, consistent with feasibility-oriented methodological studies.

## Results

The inhibitory effects of autologous platelet concentrates were evaluated with particular focus on inhibition zone detectability across different culture media. Two anaerobic periodontal pathogens, *P. gingivalis* and *P. intermedia*, were investigated. Detectability outcomes and inhibition zone magnitude were analyzed separately, as these represent distinct methodological endpoints. Inter-observer agreement for inhibition zone measurements was excellent (mean absolute difference 1.07 ± 0.82 mm; *r* = 0.998, *p* < 0.001), with identical results for plates without detectable inhibition, confirming high reproducibility of the measurement procedure.

### Inhibition zone expression of *Porphyromonas gingivalis*

For *P. gingivalis*, inhibition assays were performed on BBA and TSASB, as preliminary experiments indicated that these media provided sufficient growth and reliable inhibition zone detection for this species. Measurable inhibition zones were observed on both media; however, marked medium-dependent differences in inhibition zone expression were detected.

When pooling all evaluable inhibition zones across PRP, PRF and i-PRF, BBA demonstrated significantly larger measurable inhibition zones compared with TSASB (Mann–Whitney U test, *p* = 0.0006). Median inhibition zone diameter was 8.18 mm on BBA (*n* = 8) compared with 1.60 mm on TSASB (*n* = 9). Distribution analysis revealed a wider interquartile range on BBA (IQR 5.10–11.44 mm; range 3.41–13.16 mm) compared with TSASB (IQR 1.41–2.69 mm; range 1.03–4.91 mm). Effect size analysis demonstrated a large medium-dependent effect (Cliff’s δ = 0.92), indicating a strong matrix influence on measurable inhibition zone expression. Detectability analysis showed measurable inhibition in 53.3% of BBA assays and 60.0% of TSASB assays.

Comparison of detectability between media revealed no statistically significant difference in the proportion of non-measurable (NN) outcomes (Fisher’s exact test, *p* = 1.0), indicating that the observed medium effect for *P. gingivalis* was primarily driven by differences in inhibition zone magnitude rather than assay failure rates.

**Fig. 2:**
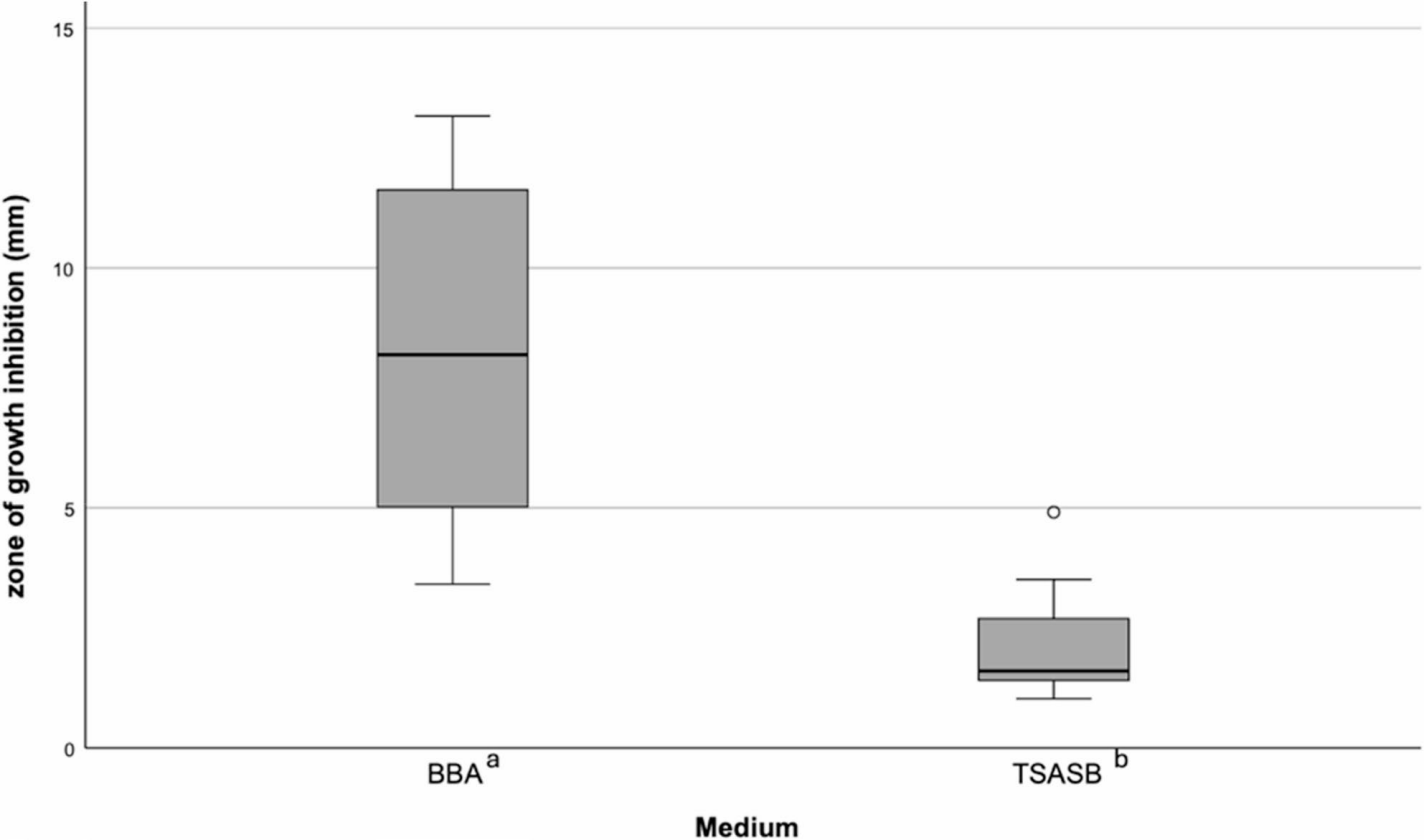
Comparison of inhibition zone expression of *P. gingivalis* on BBA and TSASB. Only those inhibition zones with a clearly defined border between growth and no growth were included. Different letters indicate statistically significant differences between culture media (Mann–Whitney U test, *p* < 0.05), whereas identical letters indicate no statistically significant difference

Only measurable inhibition zones were included in quantitative analyses, whereas non-measurable outcomes (due to indistinct inhibition borders) were excluded from zone size comparison but considered in qualitative detectability assessment. The distribution of inhibition zone diameters for *P. gingivalis* on BBA and TSASB is illustrated in Figure 2.

### Inhibition zone expression of *Prevotella intermedia*

For *P. intermedia*, measurable inhibition was reproducibly detected on BBA and MHB agar. Statistical comparison between BBA (*n* = 8) and MHB (*n* = 15) did not reveal a significant difference in measurable inhibition zone size (Mann–Whitney U test, *p* = 0.77).

Median inhibition zone diameter was 4.58 mm on BBA and 5.04 mm on MHB. Interquartile ranges were comparable between BBA (IQR 2.55–9.78 mm; range 1.61–15.32 mm) and MHB (IQR 2.59–8.17 mm; range 0–15.05 mm). Effect size analysis indicated only a trivial difference between media (Cliff’s δ = 0.08), suggesting comparable assay behavior under these conditions.

In contrast, TSASB produced no measurable inhibition zones for *P. intermedia* in any donor (all measurements 0 mm), indicating absence of detectable inhibition under the given assay conditions. Consequently, TSASB was not included in the boxplot comparison. Detectability analysis demonstrated measurable inhibition in 53.3% of BBA assays and 100% of MHB assays for *P. intermedia*.

Importantly, detectability differed significantly between BBA and MHB. While 7/15 BBA assays were classified as non-measurable (NN), no NN outcomes occurred on MHB (0/15), resulting in a statistically significant difference in inhibition zone detectability (Fisher’s exact test, *p* = 0.006).

**Fig. 3:**
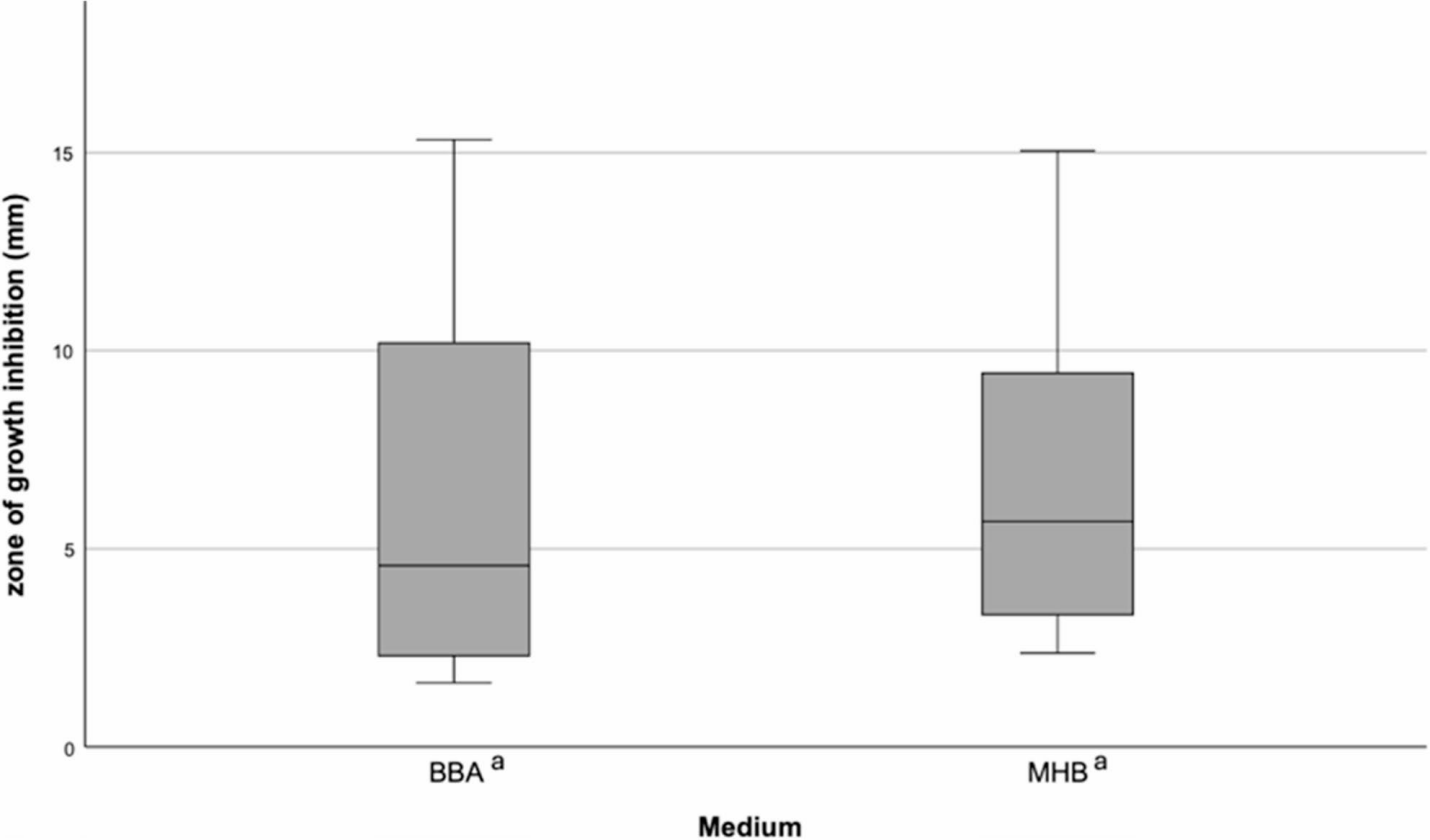
Comparison of inhibition zone expression of *P. intermedia* on BBA and MHB. TSASB is not displayed, as no measurable inhibition zones were obtained on this medium in any donor, resulting in 0% detectability. Only those inhibition zones with a clearly defined border between growth and no growth were included. Different letters indicate statistically significant differences between culture media (Mann–Whitney U test, *p* < 0.05), whereas identical letters indicate no statistically significant difference

The boxplot comparison between BBA and MHB illustrates variability in inhibition zone expression between media, with only measurable inhibition zones included in quantitative analyses. The corresponding distributions of inhibition zone diameters for *P. intermedia* are shown in Figure 3.

### Comparison of platelet concentrate preparations

**Fig. 4:**
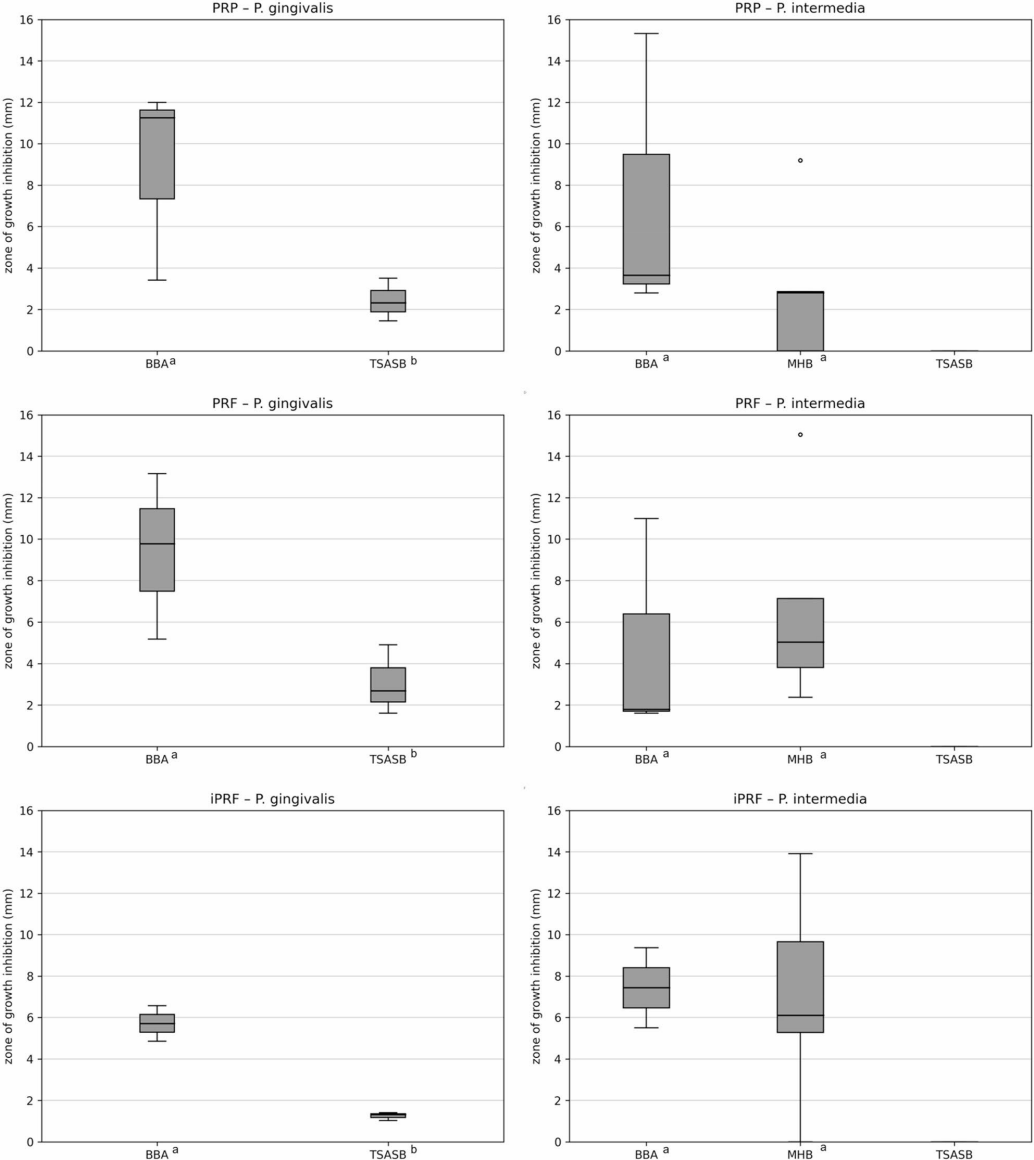
Boxplots illustrating inhibition zone diameters (mm) for the platelet preparations PRP, PRF, and i-PRF against *P. gingivalis* and *P. intermedia* on different culture media (BBA, TSASB, and MHB). Each boxplot represents the distribution of inhibition zone diameters measured for the respective preparation and medium. Different letters indicate statistically significant differences between culture media (Mann–Whitney U test, *p* < 0.05), whereas identical letters indicate no statistically significant difference. No statistically significant differences between platelet preparations were detected under the present experimental conditions. TSASB is shown for completeness but yielded no measurable inhibition zones for *P. intermedia*

When comparing the three platelet preparations (PRP, PRF, and i-PRF), inhibition zones were observed for all preparations against both *P. gingivalis* and *P. intermedia*. Overall, exploratory statistical comparisons did not reveal statistically significant differences between platelet preparations under the present experimental conditions, and these findings should be interpreted within the exploratory framework of the study. Across the tested media, PRP and PRF tended to show slightly larger inhibition zones against *P. gingivalis*, whereas i-PRF demonstrated similar or moderately smaller inhibition zones. For *P. intermedia*, inhibition zones were observed for all preparations on BBA and MHB agar, with overlapping ranges between PRP, PRF, and i-PRF. These distributions are illustrated in Fig. 4.

## Discussion

The present findings highlight that culture medium selection represents a critical methodological factor influencing inhibition zone detectability and measurable inhibition zone expression in diffusion-based antimicrobial testing. Inhibition effects were detectable for all investigated platelet preparations (PRP, PRF and i-PRF), indicating that antimicrobial activity was not restricted to a specific platelet concentrate formulation. Mueller–Hinton blood agar and BBA yielded substantially more interpretable results, whereas TSASB frequently produced absent or non-measurable inhibition zones. The absence of a measurable zone should therefore not be interpreted as true biological inactivity, but may instead reflect limitations of the assay environment, particularly in diffusion-dependent models.

For *P. gingivalis*, the medium effect was not only statistically significant (*p* = 0.0006) but also associated with a large effect size (Cliff’s δ = 0.92), indicating a pronounced matrix-dependent amplification of measurable inhibition zone expression. Importantly, detectability did not differ significantly between BBA and TSASB (*p* = 1.0), as reflected by comparable proportions of non-measurable (NN) outcomes, suggesting that the observed difference was primarily attributable to altered diffusion or matrix interaction rather than differential assay failure. In contrast, *P. intermedia* showed comparable inhibition zone distributions between BBA and MHB (*p* = 0.77; Cliff’s δ = 0.08), suggesting species-specific sensitivity to medium composition. However, detectability differed significantly between these media, as MHB demonstrated complete inhibition zone detectability (0% NN) compared with a substantial proportion of non-measurable outcomes on BBA (*p* = 0.006). Thus, for *P. intermedia*, medium selection predominantly influenced inhibition zone detectability rather than inhibition zone magnitude.

Systematic and scoping reviews repeatedly highlight that in vitro evidence on antimicrobial effects of PRF-based products is methodologically heterogeneous, with substantial variation in preparation protocols, target organisms and test conditions, limiting comparability between studies [8, 9]. These findings indicate that medium selection alone can substantially alter both inhibition detectability and measurable inhibition zone size. Notably, the direction and magnitude of this effect appear to be species-dependent, affecting either quantitative zone expression (*P. gingivalis*) or inhibition zone detectability (*P. intermedia*). Unlike classical antibiotic susceptibility testing, where disc diffusion is standardized, there is currently no comparable standard for biological preparations such as PRP/PRF/i-PRF. Consequently, findings cannot be interpreted without considering the underlying test conditions.

The consistently poor performance of TSASB in this study is likely multifactorial. First, agar diffusion assays require a stable and uniform bacterial lawn. If growth is patchy or fails—particularly under anaerobic requirements—zones become difficult or impossible to interpret, resulting in non-measurable outcomes. Second, TSASB is not designed as a standardized inhibition-test platform. Differences in agar matrix composition and blood/protein supplementation can influence diffusion characteristics and the apparent size or even visibility of inhibition zones. This is a well-known limitation of diffusion-based assays, in which the measured inhibition is not only driven by antimicrobial activity but also by physicochemical diffusion behavior within the agar [11, 23, 24]. Such effects are likely amplified when biological preparations are tested instead of purified antibiotics [2]. In the present dataset, TSASB yielded markedly smaller inhibition zones for *P. gingivalis* and no detectable inhibition for *P. intermedia*. This suggests that TSASB is unsuitable as a universal platform for diffusion-based APC inhibition assays. Importantly, this does not imply general inferiority of TSASB as a growth medium, but rather limited suitability for this specific assay context. The consistent performance of positive controls further supports the validity of the assay conditions applied in this study.

The strong medium effect observed for *P. gingivalis* is biologically plausible. This strictly anaerobic pathogen requires hemin and iron sources and is highly sensitive to environmental conditions influencing growth density and virulence regulation [25, 26]. BBA provides supplementation supporting these metabolic requirements and may facilitate dense, reproducible anaerobic growth. Under such conditions, inhibition zones may become more consistently detectable and quantitatively expressible. Thus, BBA does not enhance intrinsic antimicrobial activity but facilitates its visualization within the diffusion matrix. These considerations represent plausible mechanistic explanations but were not directly investigated in the present study.

Compared to *P. gingivalis*, *P. intermedia* demonstrated less pronounced medium dependency between BBA and MHB. The comparable inhibition zone distributions and trivial effect size suggest that this species may tolerate a broader range of basal culture conditions under anaerobic incubation. Nevertheless, the marked difference in inhibition zone detectability between BBA and MHB indicates that subtle differences in matrix composition may influence lawn stability and assay reliability even in the absence of zone size differences.

The results indicate that medium selection should be aligned with microbial growth requirements rather than applying a uniform agar system for all species. For strict anaerobes such as *P. gingivalis*, media providing appropriate heme-related supplementation appear essential for generating robust and interpretable inhibition readouts. Mueller–Hinton-based media demonstrated stable and reproducible inhibition detection and represent a pragmatic general platform. TSASB repeatedly reduced inhibition zone detectability and should be avoided in diffusion-based APC testing unless specifically validated.

In the present study, inhibition effects were detectable for all investigated platelet concentrate preparations (PRP, PRF and i-PRF), although the magnitude and detectability of inhibition zones depended on the culture medium used. Biological variability between preparations is expected due to differences in fibrin architecture, leukocyte content, platelet concentration, release kinetics, viscosity, and physical state, all of which are likely to influence diffusion behavior in agar-based systems [2, 4, 8].

In the present dataset, inhibition zones were detectable for all three preparations against both investigated pathogens, indicating that antimicrobial activity was not restricted to a specific APC formulation. Across media, PRP and PRF tended to show slightly larger inhibition zones against *P. gingivalis*, whereas i-PRF demonstrated similar or moderately smaller zones. For *P. intermedia*, inhibition zones were observed for all preparations on BBA and MHB agar, with overlapping distributions between PRP, PRF and i-PRF. Exploratory statistical comparisons between APC types did not reveal statistically significant differences under the present experimental conditions; however, due to limited statistical power, these findings should not be interpreted as evidence of equivalence between preparations. This suggests that, within the experimental conditions of the present diffusion assay, matrix-dependent assay effects exceeded potential biological differences between APC formulations.

The findings demonstrate that culture medium selection can amplify, attenuate or mask antimicrobial effects in vitro. Both qualitative outcomes (detectable vs. non-detectable) and quantitative measurements (zone size) are matrix dependent. Methodological standardization of diffusion-based APC assays is therefore a prerequisite for meaningful comparison across studies and for reliable interpretation of negative results.

This feasibility study focused exclusively on diffusion-based assays; other experimental models, including broth-based kinetics or biofilm systems, may yield different behavior patterns. Statistical analyses were performed at the assay level to investigate methodological influences on inhibition zone detectability under controlled conditions. While this approach is appropriate for the methodological focus of the study, it may introduce pseudo-replication if interpreted as reflecting donor-level biological independence. Accordingly, the results should be interpreted within an exploratory framework rather than as confirmatory evidence. Due to the limited number of donors, the study has limited statistical power to detect subtle biological differences between platelet preparations; therefore, conclusions in this regard should be interpreted as hypothesis-generating rather than definitive. In addition, the study was limited to two representative periodontal pathogens; future studies should include a broader range of microorganisms and clinical isolates to assess the generalizability of the observed matrix-dependent effects. The exclusion of non-measurable (NN) outcomes from quantitative analysis may introduce a bias toward larger observable inhibition zones. However, NN outcomes reflect limitations in detectability rather than absence of biological activity and were therefore analyzed separately as a distinct methodological endpoint. Future research should integrate complementary quantitative approaches and mechanistic investigations to further elucidate matrix-dependent diffusion behaviour in biological biomaterials.

## Conclusions

Inhibition assays of autologous platelet concentrates are highly sensitive to culture conditions, and agar medium selection substantially influences inhibition detectability and measurable inhibition zone expression.

BBA enabled significantly larger measurable inhibition zones for *P. gingivalis*, whereas Mueller–Hinton-based media provided the most robust overall assay performance. TSASB frequently reduced inhibition zone detectability and showed limited suitability for diffusion-based APC testing.

Inhibition effects were detectable for all investigated platelet preparations (PRP, PRF and i-PRF). No statistically significant differences between APC types were detected under the present experimental conditions, suggesting that matrix-dependent assay effects may exceed formulation-specific biological differences in diffusion-based APC experiments.

Culture medium selection should therefore be considered a critical methodological variable in antimicrobial evaluation of biological biomaterials. Negative findings in diffusion assays should be interpreted cautiously and in the context of the underlying assay conditions. The results should be interpreted as exploratory and hypothesis-generating rather than confirmatory.

## Data Availability

The datasets used and/or analyzed during the current study are available from the corresponding author on reasonable request.

## Abbreviations

APC: Autologous platelet concentrates
CFU: Colony-forming units
CI: Confidence interval
CLSI: Clinical and Laboratory Standards Institute
EUCAST: European Committee on Antimicrobial Susceptibility Testing
i-PRF: Injectable platelet-rich fibrin
MHB: Mueller–Hinton blood agar
NN: Non-measurable
*P. gingivalis*: *Porphyromonas gingivalis*
*P. intermedia*: *Prevotella intermedia*
PRF: Platelet-rich fibrin
PRP: Platelet-rich plasma
QC: Quality control
SD: Standard deviation
TSASB: Tryptic soy agar supplemented with blood
